# IDAC-Dose 2.2, an internal dosimetry software for diagnostic nuclear medicine based on the latest ICRP adult and paediatric reference computational phantoms

**DOI:** 10.1186/s40658-025-00774-z

**Published:** 2025-06-13

**Authors:** Martin Andersson, Keith Eckerman, Sören Mattsson

**Affiliations:** 1 Department of Radiation Physics, Sahlgrenska Cancer Center, Medical Radiation Physics, Department of Translational Medicine, University of Gothenburg, Lund University, Skåne University Hospital, Malmö, Malmö, SE-205 02 Sweden; 2 Center for Radiation Protection Knowledge, Oak Ridge National Laboratory, Oak Ridge, TN USA; 3 Medical Radiation Physics, Department of Translational Medicine, Lund University, Skåne University Hospital, Malmö, Malmö, SE-205 02 Sweden

**Keywords:** Internal dosimetry, ICRP, IDAC, DCAL, Effective dose, Radiopharmaceuticals, Diagnostic nuclear medicine

## Abstract

**Background:**

For patients investigated with radiopharmaceuticals, it is important to be able to perform valid calculations of the absorbed dose in organs and tissues. An internal dosimetry computer program, IDAC-Dose2.1, has been updated to be based on the ICRP specific absorbed fractions and computational framework of internal dose assessment for 12 adult and paediatric reference individuals given in ICRP Publication 133 and 155. The updated dosimetry software intended for nuclear medicine is named IDAC-Dose2.2. The calculations are based on radionuclide decay scheme of ICRP Publication 107. Biokinetic models can be based on up to 83 different source regions irradiating 48 target tissues, defining the effective dose as presented in ICRP Publications 60 and 103. The computer program was validated against another ICRP dosimetry software, DCAL ver. 2022, that employs the same computational framework and is used for occupational and environmental intakes of radionuclides. IDAC-Dose2.2 calculates absorbed doses to the 2 adult and 10 paediatric,15-yrs, 10-yrs, 5-yrs, 1-yr, 100 days (infant) and 0 day (new-born), sex specific ICRP reference phantoms. It has an additional sub-module which can interpolate the calculated absorbed dose and effective dose to an arbitrary age between 0 and 20 years (20 years = adult) or an arbitrary weight of 3.5–73 kg for male and 3.5–64 kg for female instead of only using the 6 fixed phantoms ages.

**Results:**

IDAC-Dose2.2 was applied on three frequently used radiopharmaceuticals: intravenously administered 2-[^18^F]FDG, orally administered ^99m^Tc-pertechnetate and ^131^I-iodide. Using the tissue weighting factors from ICRP Publication 103, the effective dose per administered activity was estimated for 2-[^18^F]FDG: 0.017mSv/MBq, 0.020 mSv/MBq, 0.029 mSv/MBq, 0.044 mSv/MBq, 0.075 mSv/MBq, 0.16 mSv/MBq 0.16 mSv/MBq for adult, 15-, 10-, 5-, 1-year old, 100 days (infant) and 0 day (newborn), respectively. Effective dose of 0.034 mSv/MBq was also calculated for 2-[^18^F]FDG to a reference person of 8-years old. For the same three radiopharmaceuticals, S-values were generated for all phantoms in IDAC-Dose2.2 and validated against the dosimetry program DCAL, showing identical results.

**Conclusions:**

The internal dosimetry program IDAC-Dose was updated to include all 12 specific sets of absorbed fractions of the ICRP adult and paediatric reference phantoms and applied to three radiopharmaceuticals for validation against DCAL and to generate improved absorbed dose estimations for preadults in diagnostic nuclear medicine. The sub-module for age or weight interpolation of absorbed doses follows the ICRP computational framework used for members of the public. IDAC-Dose2.2 will used by the ICRP for absorbed and effective dose calculations in diagnostic nuclear medicine. The results from other software, which uses the same primer data (e.g. ICRP SAF values or decay data) could deviate from those of IDAC-Dose 2.2 and published ICRP dose values if the software do not follow the ICRP computational framework for internal dosimetry. IDAC-Dose2.2 is a free software for research and available at http://www.idac-dose.org. The online version can be operated directly through a web browser and the standalone version is an executable file, which is downloaded and installed directly on the local computer.

## Background

For patients investigated with radiopharmaceuticals in nuclear medicine or persons exposed occupationally or accidently to internally distributed radionuclides, it is important to be able to perform valid estimations of the absorbed dose to organs and tissues. As direct measurements of absorbed dose are difficult or mostly impossible, the assessments rely on calculations based on measurements of the activity in various organs and tissues and its variation by time. Measurements of the excreted activity (in urine and faeces) are also possible and a way to estimate the intake of activity in occupational and accidental exposures. In nuclear medicine, the administered activity is however well known and together with frequent excretion measurement, good information on the retention of the radionuclide in the body can be obtained. Due to problems with sampling and measurements of excreta this is seldom done. Instead, as in other cases, the measured activities in organs and tissues are used to estimate the absorbed dose using models describing the time dependent variation of activity in the body and the energy deposition in the organs and tissues of interest due to emission of radiation at the decay of the radionuclide.

To be able to perform the dose estimates, the International Commission on Radiological Protection (ICRP) has developed a computational framework for internal dose assessment [1]. Two computer programs currently following this framework of the ICRP are the “Dose and Risk Calculation (DCAL)” [2] and “Internal Dose Assessed by Computer” (IDAC-Dose) [3]. DCAL version 2022 is intended for radionuclides entering the body by inhalation and ingestion during occupational and environmental exposures and has been used by the ICRP to generate radionuclide specific dose coefficients. However, DCAL version 2022 is not intended or applicable for patient dosimetry. IDAC-Dose has been created to provide ICRP with dose coefficients for patients undergoing examinations with radiopharmaceuticals in diagnostic nuclear medicine (mainly through intravenous administration, sometimes *per os* or via inhalation). Both DCAL and IDAC-Dose uses the nuclear decay data available in ICRP Publication 107, and ICRPs monoenergetic specific absorbed fractions (SAFs), for adults published in ICRP Publication 133 [4]. Both software now also include the new paediatric SAF values, which have recently been published in ICRP Publication 155 [5] including values for five different ages of children and preadults; 0 day (newborn), 1 year, 5 years, 10 years and 15 years of both sexes. The SAF values are based on the ICRP computational voxel phantoms [6,7] with underlying anatomical and physiological reference values for the 12 reference individuals presented in ICRP Publication 89 [8]. Similar computer programs developed by other groups (MIRD [9], RADAR [10], etc.) have either based the absorbed dose calculation on the ICRP Publication 89 [8] or the previous reference values given in ICRP Publication 23 [11]. In developing guidelines to limit potential stochastic effects (mainly cancer) from radiation exposure (external and internal), the ICRP defines an idealised person called the “Reference Person” and the dosimetric quantity effective dose [12,13]. To calculate the effective dose, the equivalent doses to the “Reference Male” and “Reference Female” are averaged.

The aim of this work was to update the IDAC-Dose2.1 [3] to IDAC-Dose2.2 to calculate absorbed dose for all 12 ICRP reference individuals and the 0 day (newborn) phantoms. IDAC-Dose2.2, is based on the SAF values published by the ICRP [4,5] and implements the computational framework for internal dose assessment already used by ICRP for dose calculations at occupational and public exposures [1]. The program enables calculations of absorbed dose and effective dose for the 1252 radionuclides published in ICRP Publication 107 provided that there are reliable biokinetic data for the substances. This update allows users to perform dose calculations based on their own biokinetic data for an arbitrary age of the reference person.

A second aim of this work was to continue the harmonisation the calculations of the ICRP preadult phantoms with the independent computer program DCAL ver. 2022 and validate the calculations for ^18^F, ^99m^Tc and ^131^I and also incorporate the interpolation of age or weight specific absorbed doses following the ICRP computational framework used to calculate internal dose coefficients for members of the public. Both IDAC-Dose2.2 and DCAL ver 2022 base their dose calculations on the same ICRP computational framework for internal dose assessment [1]. However, IDAC-Dose2.2 is intended for diagnostic nuclear medicine, the software includes also clinical parameters as organ specific time integrated activity coefficient and dynamic urinary bladder calculations. IDAC-Dose2.2 will be used by the ICRP Task Group on “Radiation dose to patients in diagnostic nuclear medicine” for new dose calculations for patients in future ICRP Publications, whereas the DCAL version 2022 will be used by the ICRP Task Group on “Internal dose coefficients” in their publications on dose estimates for occupational and public intake of radionuclides [1,14–17].

## Absorbed dose and effective dose

MIRD pamphlet 21 [18] defines the mean absorbed dose ($\:D$) to a target region ($\:{r}_{T}$) for a time-independent system as show in Eq. 1.

$$\:D\left({r}_{T},{T}_{D}\right)={\sum\:}_{{r}_{S}}\stackrel{\sim}{A}\left({r}_{S},{T}_{D}\right)S\left({r}_{T}\leftarrow\:{r}_{S}\right)\:\left[Gy\right]\:\:\:\:\:\:\:\:\:\:\:\:\:\:\:\:\:\:\:\left(1\right)$$

where *Ã*(*r*_*S*_, *T*_*D*_) is the time-integrated or cumulated activity (i.e., the total number of disintegrations in source region $\:{r}_{S}$ over the integration period $\:{T}_{D}$. The integration period in IDAC-Dose2.2, is in hours and $\:{T}_{D}$ is normally set to ∞. $\:S\left({r}_{T}\leftarrow\:{r}_{S}\right)$ is the mean absorbed dose in the target tissue per nuclear transformation in the source region and defined as show in Eq. 2.

$$\:S\left({r}_{T}\leftarrow\:{r}_{S}\right)={\sum\:}_{i}{\varDelta\:}_{i}\varPhi\:\left({r}_{T}\leftarrow\:{r}_{S},{E}_{i}\right)\:\left[Gy/Bq\right]\:\:\:\:\:\:\:\:\:\:\:\:\:\:\:\:\:\:\:\left(2\right)$$

where $$\:\varPhi\:\left({r}_{T}\leftarrow\:{r}_{s},{E}_{i}\right)$$ is the specific absorbed fraction (kg^− 1^) [4] of the energy of the $$\:i$$th radiation emitted within source region $$\:{r}_{s}$$ that is absorbed per mass in target region $$\:{r}_{T}$$ and $$\:{\varDelta\:}_{i}={E}_{i}{Y}_{i}$$ is the emitted energy per nuclear transformation (J $$\:{\left(Bq\:s\right)}^{-1}$$) [18]. Here, $$\:{Y}_{i}$$$$\:{\left(Bq\:s\right)}^{-1}$$ is the yield of the $$\:i$$th radiation per nuclear transformation and $$\:{E}_{i}$$ is the emitted energy (J). Each type of emitted radiation is considered, that is the summation extends over electrons, alpha particles, photons and an integral over the beta spectrum.

The effective dose (E) is a radiation protection quantity defined by the ICRP to estimate the risk that radiation will cause detrimental stochastic effects in the reference population. ICRP has revised the tissue weighting factors twice and, in the latest version presented in ICRP Publication 103 [12], the risk to the Reference Person is calculated as show in Eq. 3.

3 $$\:E={\sum\:}_{T}{w}_{T}{\sum\:}_{R}\frac{{w}_{R}{D}_{R}{\left({r}_{T},T\right)}_{Ref.\:\:male}+{w}_{R}{D}_{R}{\left({r}_{T},T\right)}_{Ref.\:\:female}}{2}\left[Sv\right]\:\:\:\:\:\:\:\:\:\:\:\:\:\:\:\:\:\:\:$$

where $\:{w}_{R}$ is the radiation weighting factor for radiation type $\:R$, which in this case is 1 for photons and electrons and 20 for alphas; $\:{w}_{T}$ is the tissue weighting factor, which is the relative contribution from organ and tissue *T* to the total detriment of stochastic effects caused by ionising radiation$\:;$*D*_*R*_*(r*_*T*_,*T)*_*Ref, male*_ and *D*_*R*_*(r*_*T*_,*T)*_*Ref, female*_ are the mean absorbed doses to the target region for the Reference Male and Reference Female, respectively, for each radiation type *R*.

### Estimation of organ specific time integrated activity coefficient

**Fig. 1:**
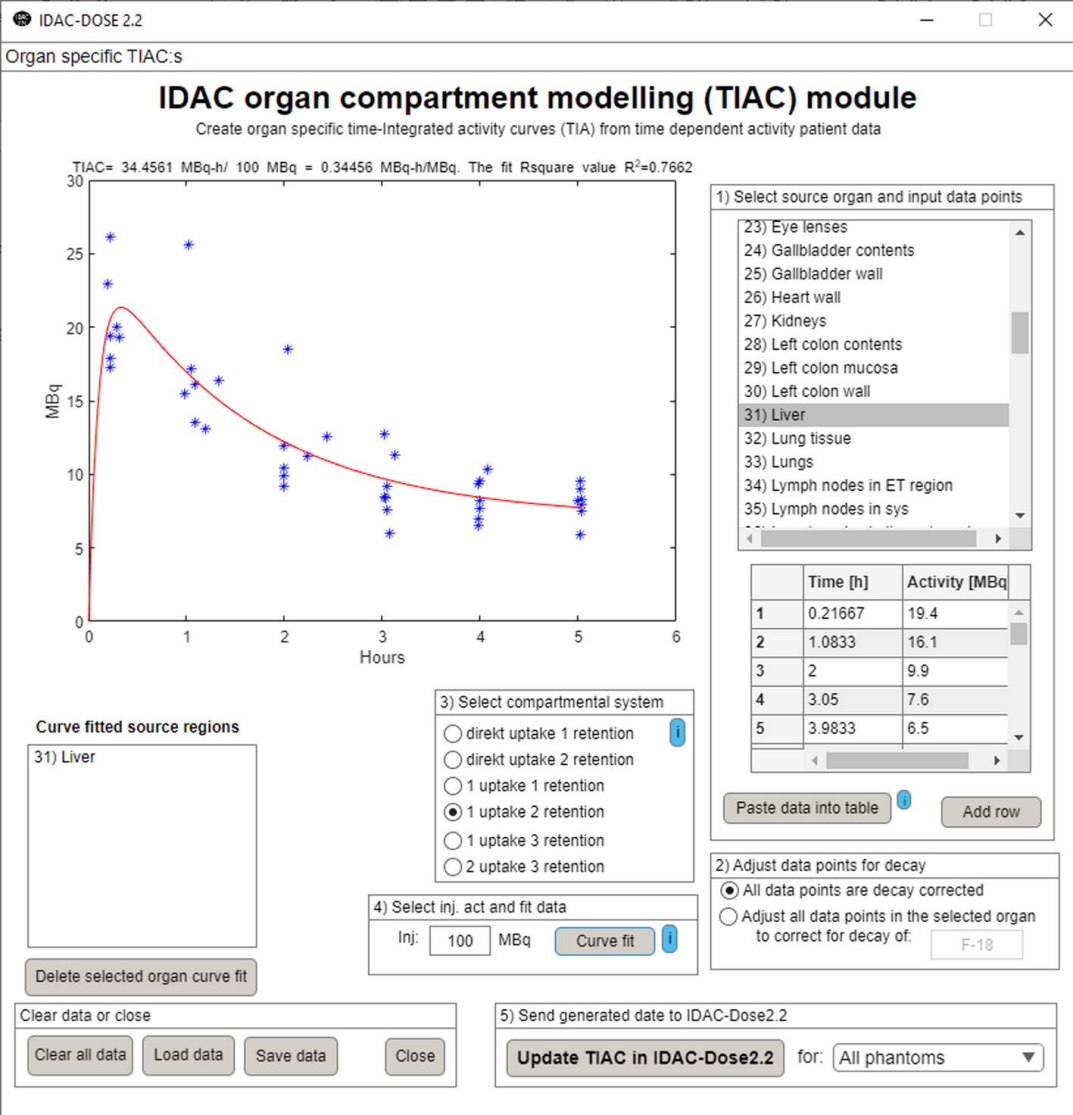
A compartment based time integration activity coefficients calculation based on a biokinetic model for seven [18 F]-Flutemetamol patients for the source region “Liver”

To make IDAC-Dose2.2 more useful, a compartment based time integrated activity coefficient (TIAC) modelling tool has been added to the software. This organ curve fitter creates a retention curve fit based on activity data points gathered at different time points. The sub-module can perform retention curve fitting to get TIAC: s for all source organs. However, the type of compartment fitting which the software will apply to the data set has to be decided manually out of five different compartmental systems (including direct, 1 or 2 uptake and selecting 1 to 3 retention components). By adding the administered activity, the cumulated activity per injected activity (normalized time activity curves) can be calculated and directly uploaded as input values for the absorbed dose calculations. This can be performed directly for all organs and phantoms. The organ retention fitting tool is shown in Fig. 1. where it is applied on a biokinetic model for seven [^18^F]-Flutemetamol patients.

### Dynamic urinary bladder calculations

**Fig. 2:**
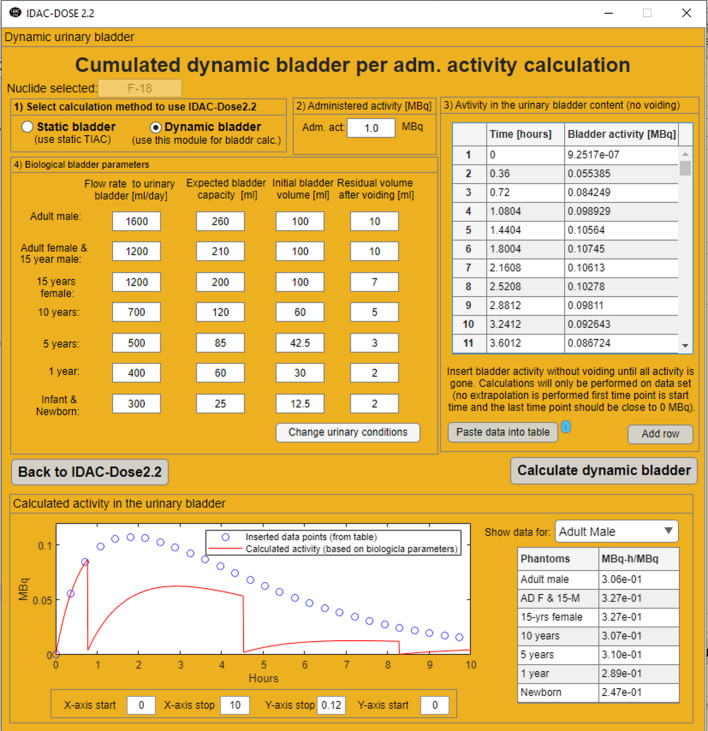
Dynamic bladder calculations, based on biological parameters for intravenous administered activity of 2-[^18^F]FDG [29]. The blue line is time dependent activity in the urinary bladder content assuming no voiding and the red line is with dynamic bladder calculation

As IDAC-Dose2.2 is developed for biokinetic compartment models which are time dependent, there is also a possibility to preform dynamic urinary bladder calculations where the S-value from the urinary bladder content to the urinary bladder wall changes with respect to volume of the content. Dynamic urinary bladder calculations are nothing new. Cloutier et al. [19] in 1973 calculated absorbed doses from photons to the uterus and the foetus using a dynamic urinary female bladder. The most used method of performing dynamic urinary bladder calculations was the dynamic model of Thomas et al. [20,21], which was further developed with dynamic S-values by Andersson et al. [22]. The sub-module for dynamic bladder calculations uses the method of the adult S-values presented by Andersson et al. [22] but uses the ICRP reference value to also include dynamic S-values for all reference phantoms. Instead of using a fix volume and a cumulated activity to the urinary bladder instead the activity time dependent compartment models can be used together to estimate biological parameters as, flow rate, expected voiding capacity, residual and initial bladder content. The sub-module also allows hydration, as for some examinations patients are drinking liquids, as an absorbed dose reduction measure, to speed up the voiding. The sub-module only calculates the absorbed dose from the urinary bladder content to the urinary bladder wall from time dependent activity data assuming no voiding. For the other target organs, the absorbed doses are calculated by using the ICRP SAF-values and the total cumulated activity in the urinary bladder content. The dynamic bladder module is presented in Fig. 2 on the ICRP biokinetic compartment model for orally administered activity of ^131^I-iodide together with the ICRP reference biological parameters [8].

## Materials and methods

### Software code and validation

**Fig. 3:**
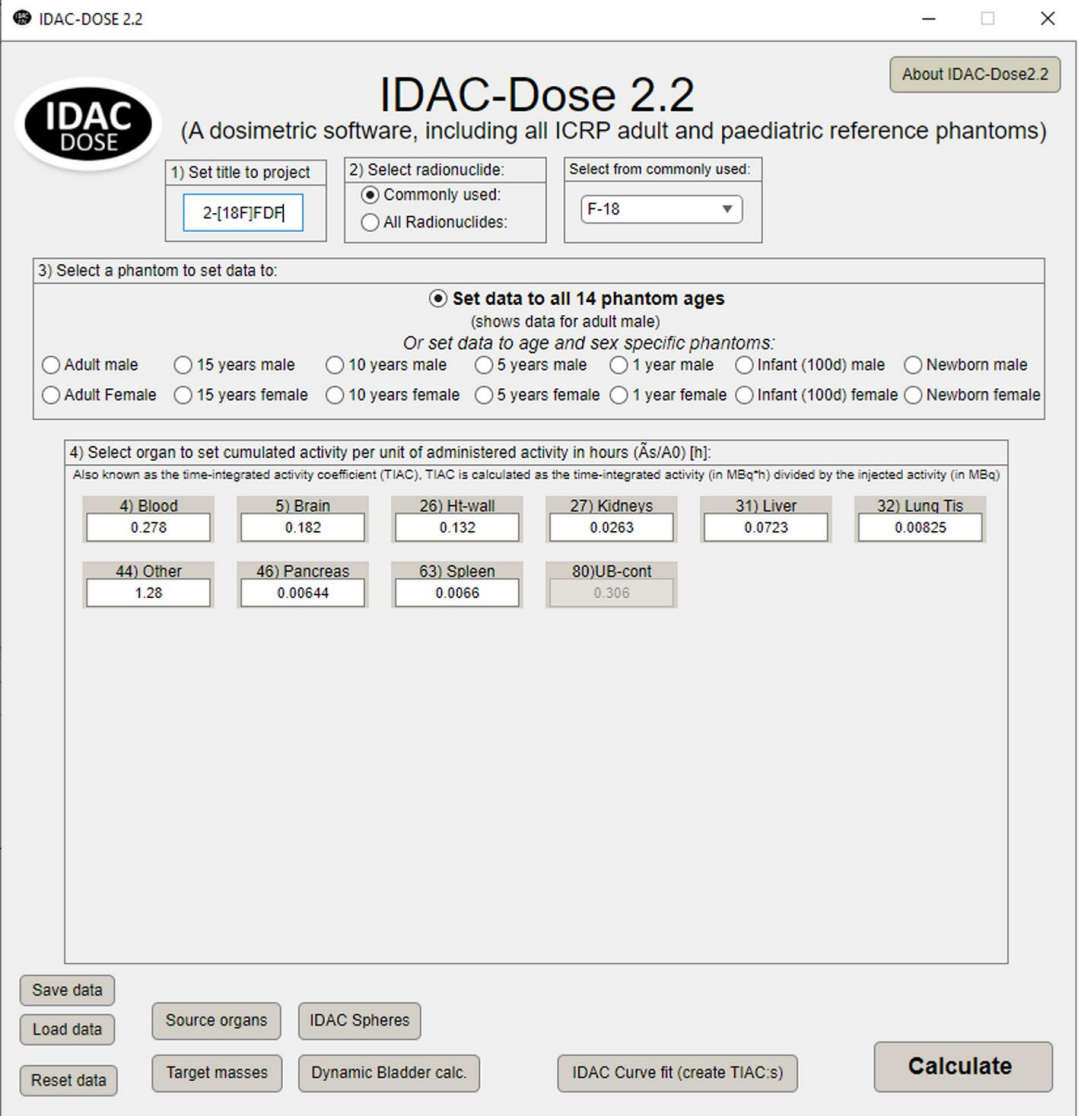
Example of the IDAC-Dose2.2 interface. Radionuclide ^18^F has been selected as an example and the cumulated activity per administered activity given in hours from the biokinetic model of 2-[^18^F]FDG in Kamp et al. [29]

The IDAC-Dose2.2 program was developed in MATLAB (MathWorks, Natick, MA, USA) and compiled as a standalone program, including a graphical user interface. The S-values generated for the radionuclides ^18^F, ^99m^Tc and ^131^I including radiation types of alpha, beta, and gamma decays were benchmarked against DCAL ver 2022. The updated version presented in Fig. 3, including a new feature with a popup window for 17 commonly used radionuclides in nuclear medicine.

### Input data for IDAC-Dose2.2

The input data for the dose calculation part of the program is the total number of disintegrations (or cumulated activity) in a source region ($\:{r}_{s}$) divided by the administered activity (Eq. 4):

$$\:\frac{\stackrel{\sim}{A}}{{A}_{0}}=\frac{{\int\:}_{0}^{{\text{T}}_{D}}A\left({r}_{S},t\right)dt}{{A}_{0}}\left[h\right]\:\left(4\right)$$

where $\:A\left({r}_{s},t\right)$ is the activity of the radiopharmaceutical in source region $\:{r}_{s}$ at time $\:t$, and $\:{A}_{0}$ is the administered activity. $\:A\left({r}_{s},t\right)$ and $\:{A}_{0}$ should be given in MBq and the integration step must be in hours. In Fig. 3, the cumulated activities per administered activity in various organs and tissues are presented for ^18^F-fluorodeoxyglucose, 2-deoxy-2-fluoro-D-glucose (FDG). IDAC-Dose2.2 calculates absorbed doses to all 47 target regions defined in ICRP Publ. 133 or Publ. 155 [4,5] and two different sets of tissue weighting factors [12,23] to estimate the effective dose. The updated IDAC-Dose2.2 includes a new target tissue called “Remainder tissues”, which can be used for other organs and tissues not listed as a target organ or tissue. The absorbed dose to these tissues is defined as the arithmetic mean dose of 13 organs and tissues as indicated in the footnote to Table 3 of ICRP Publication 103 [12] and given a tissue weighting factor of 0.12. The results are presented in terms of absorbed dose per administered activity (mGy/MBq) and effective dose per administered activity (mSv/MBq).

### Decay scheme

For dosimetric calculations, ICRP Publication 107 [24] provides information on physical half-lives, decay chains, and the yields and energies of radiation emitted in the nuclear transformations of 1252 radionuclides of 97 elements. This database supersedes the data of ICRP Publication 38 [25] and will be used in future ICRP publications on dose coefficients for the intake or exposure to radionuclides in the workplace, environment, and nuclear medicine. IDAC-Dose2.2 incorporates the nuclear decay data for all radionuclides and performs dose calculations for photons, electrons, and alpha particles. IDAC-Dose2.2 also incorporates the Publication 107 beta spectra of 955 radionuclides and calculates radionuclide-specific $\:{S}_{beta}\left({r}_{T}\leftarrow\:{r}_{S}\right)$ values. The $\:{S}_{beta}\left({r}_{T}\leftarrow\:{r}_{S}\right)$ values are generated by integration over the beta particle energy distribution; i.e., the summation in Eq. 2 is replaced with an integral.

### Source regions

The computer program has 83 different source regions, 79 of which were addressed in SAF tables of ICRP Publication 133 and 155 [4,5]. Three of the source regions not included in the SAF tables are the source regions “Total body”, “Total body excluding content”, and “Other”, which are potential source regions in biokinetic models. The source region “Blood” represents the blood circulating in the body. If in a biokinetic model an organ/tissue has an activity contribution of circulating blood, then the circulating blood in other source regions must be subtracted, to avoid a decay being registered in more than one source region. For biokinetic models that do not include the circulating blood as a source region, the program will automatically account for this by adding parts of the SAF values for the source region Blood (e.g., large vessels and heart content in the source region Other) following the recommended reference values for regional blood volumes in children and adolescents [26], otherwise this part of the phantoms would not be taken into account in the absorbed dose calculation. The source region “Other”, previously called Remainder [27], is a combination of several source regions and has no explicit SAF values, in ICRP Publication 133 [4]. The Other resembles all soft tissues not included in a biokinetic model, meaning that there is no assumed enhanced uptake of the radiopharmaceutical in these regions, making it specific for each biokinetic model. The SAF values for the source region are calculated as Eq. 5:

$$\:\varPhi\:\left({r}_{T}\leftarrow\:{r}_{Other}\right)=\frac{1}{{M}_{Other}}{\sum\:}_{{r}_{S,O}}{M}_{{r}_{S,O}}\varPhi\:\left({r}_{T}\leftarrow\:{r}_{S,R}\right)\:\left[kg\right]\:\left(5\right)$$

where *r*_*S, O*_ is the source region *S* included in the source region Other (*O*), and $\:{M}_{Other}$ and $\:{M}_{{r}_{S,O}}$ are the total mass of the Other and each individual source region included in the source region Other.

### Target regions

**Fig. 4:**
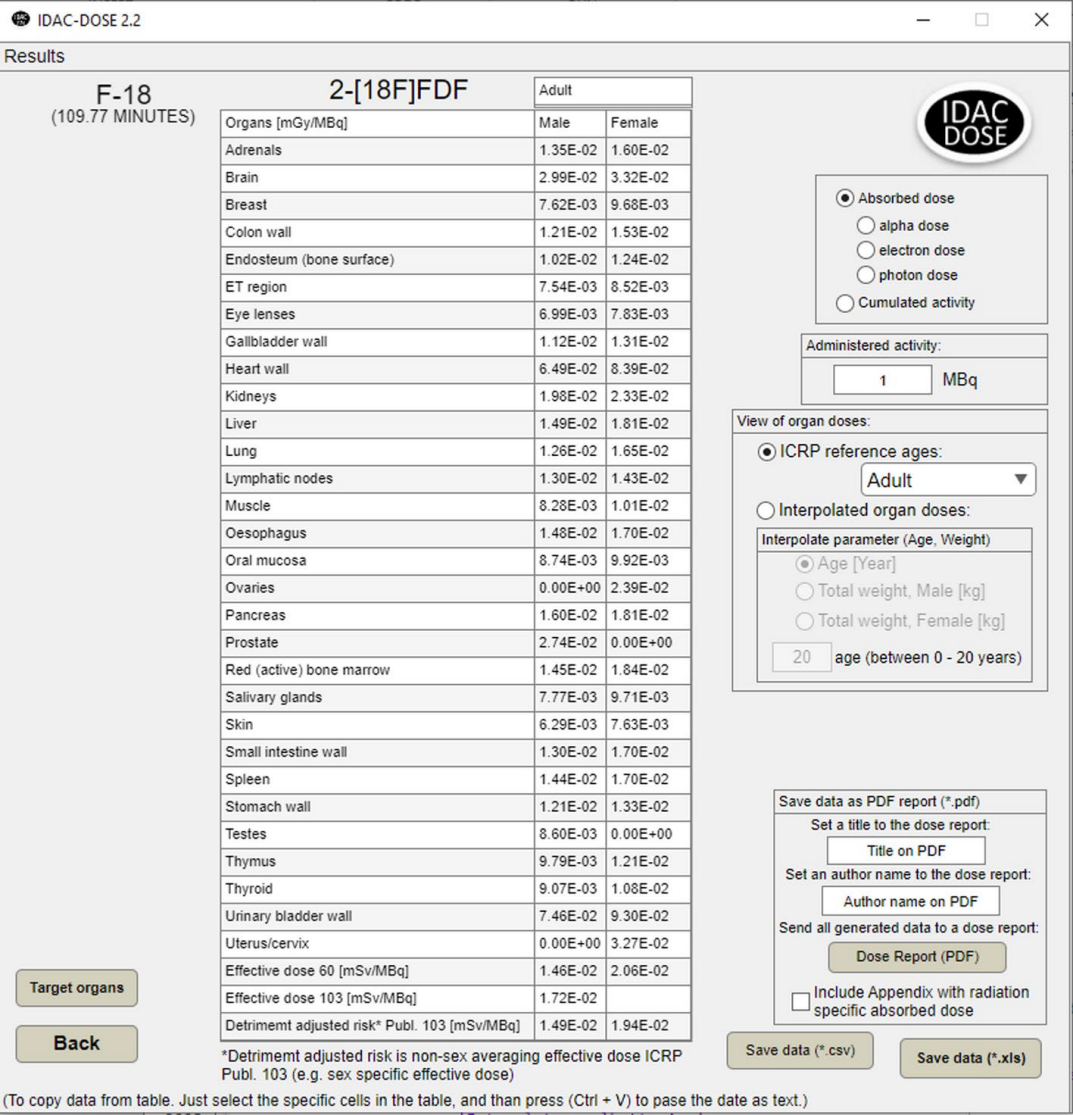
Using IDAC-Dose2.2 to calculate absorbed dose. Absorbed dose (in mGy) for the organs and tissues included in the list of tissues with a weighting factor in ICRP Publication 103 and the effective dose as defined in ICRP Publications 60 and 103 (in mSv) together with the absorbed dose to the lens of the eye for the biokinetic model of the intravenous administration of 1 MBq of 2-[^18^F]FDG based on the biokinetic model presented by Kamp et al. [29]

Forty-eight different target regions are defined in ICRP Publication 133 and 155 [4,5]. IDAC-Dose2.2 calculates the absorbed dose to all regions. If their mass has been altered to adjust for self-irradiation, an additional calculation of absorbed dose to other organs and effective dose will be performed. Figure 4 shows the absorbed doses for organs and tissues for which tissue weighting factors are given in ICRP Publication 103 [12]. As an extension not included in the ICRP computational framework does IDAC-Dose2.2 also an detriment adjusted radiation dose. In this case is the detriment adjustment that the equivalent doses are not sex averaged, and the effective dose is calculated for each sex separately. However, there is a possibility to include all 48 target regions and the effective dose as defined in ICRP Publications 60 and 103 in the result list.

### Calculations of ICRP reference infant (100 days old)

The ICRP gives SAF values for adult, 15- 10- 5-, 1 and 0 day (newborn). To cover a more clinically important age group ICRP will instead, provide the absorbed and effective dose to a reference infant individuals resembling a baby at an age of 100 days. To generate the S-coefficient for a reference infant at day 100, the S-coefficient from the 1-year-old individual and the 0 day are used. The S-coefficients calculations for infant are given in Eq. 2.16 and Eq. 2.17 in ICRP Publ. 155 [5] and the calculation can be reduced for a 100-day infant to:

6 $$\:{S\left({r}_{T}\leftarrow\:{r}_{S}\right)}_{100\:days}=0.6528*{(S\left({r}_{T}\leftarrow\:{r}_{S}\right)}_{1\:yr}-{S\left({r}_{T}\leftarrow\:{r}_{S}\right)}_{0\:day})+{S\left({r}_{T}\leftarrow\:{r}_{S}\right)}_{0\:day}\:\:\:$$

where $\:{S\left({r}_{T}\leftarrow\:{r}_{S}\right)}_{100\:days}$ is the S-coefficient for 100 days and $\:{S\left({r}_{T}\leftarrow\:{r}_{S}\right)}_{1\:yr}$ and $\:{S\left({r}_{T}\leftarrow\:{r}_{S}\right)}_{0\:day}$ are the S-coefficient for 1 year old and 0 day, respectively.

### Specific dosimetry

IDAC-Dose2.2 is created for diagnostic nuclear medicine reference dosimetry based on standardised anatomical and biokinetic models for patients. In addition, there are also two tools in the software created for patient-specific dosimetry, which allows adjustments for the activity distribution within an organ or tissue and the ability to estimate absorbed doses to anatomical spherical volumes. These two additions are important for dosimetry in therapy with radiopharmaceuticals. The software includes an interpolation sub-module, which provides the possibility to estimate age and weight dependent calculations for individuals who differ from the ICRP reference phantoms. The sub-module which calculates absorbed dose to 21 separate types of different tissue materials of spherical structure, ranging from 0.01 cm^3^ to 3000 cm^3^ have been updated to also include the separate absorbed dose contribution from photons, electrons and alpha particles to the absorbed dose. This gives an improved understanding of the radiation dose contribution from alpha particles, electrons and photons for all 1252 radionuclides, included in the decay database [24]. It is also possible to include the progenies for 587 different radionuclides in the sphere calculations using the Bateman equations for serial decay [28], while in the previous version only nine alpha emitting radionuclides were included. The 12 reference computational phantoms are *all connected to an reference age where 3 months*,* 1 year*,* 5 years*,* 10 years*,* 15 years and adult corresponds to an age range of 0 to 12 mo*,* from 1 y to 2 y*,* more than 2 y to 7 y*,* more than 7 y to 12 y*,* more than 12 y to 17 y*,* more than 17 y*,* respectively. However*,* there is a possibility to perform age specific calculations in IDAC-Dose2.2.* A new sub-module is included in IDAC-Dose2.2, which interpolates absorbed doses for a specific age or a specific total body weight. The sub-module uses all the calculated absorbed organ doses from the 12 reference computational phantoms and interpolates based on age or weight. Therefore, the range of this interpolation is limited to be valid between the newborn phantoms (100 days and 3.5 kg) and the adult phantoms (20 years = adult or male 73 kg; female 64 kg). The effective dose is calculated based on the age or total weight absorbed dose values.

### Examples of dose calculations with IDAC-Dose2.2

Absorbed doses were calculated for three radiopharmaceuticals: 2-[^18^F]FDG, ^99m^Tc-pertechnetate, and ^131^I-iodide, which are all frequently used clinically.

### 2-[^18^F]FDG

Absorbed dose per unit administered activity was calculated for all 14 reference individuals for an 2-[^18^F]FDG examination using the revised biokinetic model by Kamp et al. [29], which is proposed to the next ICRP biokinetic model. Different age and sex biological parameters were used for the dynamic bladder calculations. Absorbed doses were also calculated for an 8-year-old reference person (corresponding to a total weight of 26 kg for both male and female) by using the absorbed dose from all phantoms for preadults members of the public and used for calculating internal dose coefficients for public intake of radionuclides.

### ^99m^Tc-pertechnetate

The dose calculations for the orally administered activity of ^99m^Tc-pertechnetate were performed using a slight modification of the biokinetic model by Leggett and Giussani [30] for systemic technetium in adults, assuming no uptake in the liver. The biokinetic model by Leggett and Giussani is not based solely on data for pertechnetate, as clinical data often show minor uptake in the liver due to the uptake of ^99^Mo-molybdate present in small amounts in the generator produced pertechnetate. Therefore, the reported uptake in the liver is included in the source organ “Other”, which is also in accordance with the pertechnetate model presented in ICRP Publication 53 [31]. For the preadult biokinetic modelling also age and sex specific excretion coefficients for the human alimentary tract model [32] were applied together with age specific biological parameters for the dynamic urinary bladder [27,33]. Absorbed doses were also calculated for a reference male person with a total weight of 68 kg.

### ^131^I-iodide

Absorbed doses were calculated for an orally administered activity of ^131^I-iodide based on the age-specific biokinetic model for iodide presented by Leggett [34,35]. The biokinetic model for iodide for occupational exposure is given in ICRP Publication 134 [14] and for diagnostic nuclear medicine a modified version was presented in ICRP Publication 128 [27] and used together with the stylized phantoms. For the biokinetic modelling age and sex specific excretion coefficients for the human alimentary tract model [31] were applied together with age specific biological parameters for the dynamic urinary bladder [27,33]. Absorbed doses were also calculated for a reference female person with a total weight of 45 kg.

## Results

The S-values generated with IDAC-Dose2.2 and DCAL ver. 2022 showed identical results within three figures for all three radionuclides, ^18^F, ^99m^Tc and ^131^I. The effective doses per unit administered activity for adult, 15- 10-, 5-, 1-year, 100 days (infant) and 0 day (newborn) and 2-[^18^F]FDG [29], using the tissue weighting factors from ICRP Publication 103, were calculated to 0.017 mSv/MBq, 0.020 mSv/MBq, 0.029 mSv/MBq, 0.044 mSv/MBq, 0.075 mSv/MBq 0.16 mSv/MBq and 0.16 mSv/MBq respectively. The effective dose per unit administered activity was also calculated for 2-[^18^F]FDG to 0.034 mSv/MBq for a “interpolated” individual of 8 years old. Absorbed doses for the reference phantoms are presented in Table 1 for the target organs given a tissue weighting factor in ICRP Publication 103 with the absorbed dose data for the lens of the eye. For intravenous administration of 2-[^18^F]FDG, the highest absorbed dose is received by the urinary bladder wall and heart wall for all 14 reference individuals and phantoms.

**Table 1a:** Absorbed dose per unit of administered 2-[^18^F]FDG activity calculated with IDAC-Dose2.2

Organ	2-[^18^F]FDG							
	Adult		15 years old		10 years old		5 years old	
	Male	Female	Male	Female	Male	Female	Male	Female
Adrenals	1.4E-02	1.6E-02	1.2E-02	1.3E-02	2.0E-02	2.0E-02	3.4E-02	3.4E-02
Brain	3.0E-02	3.3E-02	3.5E-02	3.8E-02	3.7E-02	4.1E-02	4.1E-02	4.5E-02
Breast	7.6E-03	9.7E-03	9.2E-03	1.0E-02	1.4E-02	1.4E-02	2.5E-02	2.4E-02
Colon wall	1.2E-02	1.5E-02	1.5E-02	1.4E-02	2.2E-02	2.2E-02	3.7E-02	3.5E-02
Endosteum (bone surface)	1.0E-02	1.2E-02	1.6E-02	1.6E-02	2.4E-02	2.4E-02	4.5E-02	4.4E-02
ET region	7.5E-03	8.5E-03	2.0E-02	2.0E-02	2.5E-02	2.5E-02	2.9E-02	2.9E-02
Eye lenses	7.0E-03	7.8E-03	1.4E-02	1.4E-02	2.0E-02	2.0E-02	2.5E-02	2.5E-02
Gallbladder wall	1.1E-02	1.3E-02	1.1E-02	1.4E-02	1.8E-02	1.8E-02	2.9E-02	2.9E-02
Heart wall	6.5E-02	8.4E-02	8.3E-02	9.1E-02	1.4E-01	1.4E-01	2.2E-01	2.2E-01
Kidneys	2.0E-02	2.3E-02	2.2E-02	2.4E-02	3.2E-02	3.2E-02	5.3E-02	5.3E-02
Liver	1.5E-02	1.8E-02	1.7E-02	2.0E-02	2.8E-02	2.8E-02	4.2E-02	4.2E-02
Lungs	1.3E-02	1.6E-02	1.2E-02	1.4E-02	2.0E-02	2.0E-02	3.0E-02	3.0E-02
Lymphatic nodes	1.3E-02	1.4E-02	1.2E-02	1.2E-02	1.8E-02	1.8E-02	3.0E-02	3.0E-02
Muscle	8.3E-03	1.0E-02	9.7E-03	1.0E-02	1.6E-02	1.6E-02	2.6E-02	2.5E-02
Oesophagus	1.5E-02	1.7E-02	1.6E-02	1.6E-02	2.5E-02	2.5E-02	4.0E-02	4.0E-02
Oral mucosa	8.8E-03	9.9E-03	2.0E-02	2.0E-02	2.7E-02	2.7E-02	3.3E-02	3.3E-02
Ovaries	---	2.4E-02	---	3.8E-02	---	5.0E-02	---	7.4E-02
Pancreas	1.6E-02	1.8E-02	1.6E-02	1.9E-02	2.8E-02	2.8E-02	4.5E-02	4.5E-02
Prostate	2.7E-02	---	3.1E-02	---	5.3E-02	---	7.5E-02	---
Red (active) bone marrow	1.5E-02	1.8E-02	1.8E-02	1.9E-02	2.5E-02	2.5E-02	3.9E-02	3.8E-02
Salivary glands	7.8E-03	9.7E-03	2.1E-02	1.9E-02	2.5E-02	2.5E-02	3.3E-02	3.3E-02
Skin	6.3E-03	7.7E-03	8.0E-03	8.6E-03	1.3E-02	1.3E-02	2.2E-02	2.2E-02
Small intestine wall	1.3E-02	1.7E-02	1.3E-02	1.3E-02	1.8E-02	1.9E-02	3.3E-02	3.5E-02
Spleen	1.4E-02	1.7E-02	1.4E-02	1.6E-02	2.3E-02	2.3E-02	3.8E-02	3.8E-02
Stomach wall	1.2E-02	1.3E-02	1.1E-02	1.3E-02	1.8E-02	1.8E-02	3.1E-02	3.1E-02
Testes	8.6E-03	---	1.9E-02	---	2.4E-02	---	3.9E-02	---
Thymus	9.8E-03	1.2E-02	1.4E-02	1.4E-02	2.2E-02	2.2E-02	3.6E-02	3.6E-02
Thyroid	9.1E-03	1.1E-02	1.3E-02	1.3E-02	1.8E-02	1.8E-02	3.2E-02	3.2E-02
Urinary bladder wall	7.5E-02	9.2E-02	9.1E-02	9.2E-02	1.5E-01	1.5E-01	1.9E-01	1.9E-01
Uterus/cervix	---	3.3E-02	---	9.0E-02	---	1.3E-01	---	8.7E-02
Effective dose (ICRP publ. 60) [mSv/MBq]	1.5E-02	2.1E-02	1.9E-02	2.3E-02	2.8E-02	3.3E-02	4.7E-02	5.4E-02
Effective dose (ICRP publ. 103) [mSv/MBq]	1.7E-02		2.0E-02		2.9E-02		4.4E-02	
Detriment adjusted radiation dose* (ICRP publ. 103) [mSv/MBq]	1.5E-02	1.9E-02	1.8E-02	2.1E-02	2.8E-02	3.0E-02	4.2E-02	4.5E-02

*Detriment adjusted radiation dose is non-sex averaging effective dose, e.g. sex specific effective dose

Data in Table 1a are given in mGy/MBq unless otherwise noted. Effective dose was calculated using the two sets of tissue weighting factors given in ICRP Publications 60 and 103

**Table 1b:** Absorbed dose per unit of administered 2-[^18^F]FDG activity calculated with IDAC-Dose2.2

Organ	2-[^18^F]FDG							
	1 year old		Infant (100 days)		Newborn (0 day)		Interpol. 8 years old	
	Male	Female	Male	Female	Male	Female	Male	Female
Adrenals	6.5E-02	6.5E-02	1.4E-01	1.4E-01	1.4E-01	1.4E-01	2.5E-02	2.5E-02
Brain	5.6E-02	5.6E-02	1.2E-01	1.2E-01	1.2E-01	1.2E-01	3.8E-02	4.3E-02
Breast	4.2E-02	4.2E-02	1.1E-01	1.1E-01	1.1E-01	1.1E-01	1.8E-02	1.7E-02
Colon wall	7.2E-02	7.1E-02	1.6E-01	1.5E-01	1.6E-01	1.5E-01	2.7E-02	2.6E-02
Endosteum (bone surface)	8.9E-02	8.9E-02	1.7E-01	1.7E-01	1.7E-01	1.7E-01	3.0E-02	3.0E-02
ET region	4.0E-02	4.0E-02	9.9E-02	9.9E-02	9.9E-02	9.9E-02	2.6E-02	2.6E-02
Eye lenses	3.7E-02	3.7E-02	1.0E-01	1.0E-01	1.0E-01	1.0E-01	2.2E-02	2.1E-02
Gallbladder wall	4.9E-02	5.0E-02	1.2E-01	1.2E-01	1.2E-01	1.2E-01	2.1E-02	2.1E-02
Heart wall	3.9E-01	3.9E-01	9.2E-01	9.2E-01	9.2E-01	9.2E-01	1.6E-01	1.6E-01
Kidneys	8.9E-02	8.9E-02	2.1E-01	2.1E-01	2.1E-01	2.1E-01	3.9E-02	3.8E-02
Liver	7.4E-02	7.4E-02	1.6E-01	1.6E-01	1.6E-01	1.6E-01	3.3E-02	3.2E-02
Lungs	5.8E-02	5.8E-02	1.4E-01	1.4E-01	1.4E-01	1.4E-01	2.3E-02	2.3E-02
Lymphatic nodes	5.1E-02	5.1E-02	1.3E-01	1.3E-01	1.3E-01	1.3E-01	2.2E-02	2.1E-02
Muscle	4.8E-02	4.8E-02	1.3E-01	1.3E-01	1.3E-01	1.3E-01	1.9E-02	1.9E-02
Oesophagus	6.7E-02	6.7E-02	1.5E-01	1.5E-01	1.5E-01	1.5E-01	3.0E-02	3.0E-02
Oral mucosa	4.9E-02	5.0E-02	1.5E-01	1.5E-01	1.5E-01	1.5E-01	2.9E-02	2.9E-02
Ovaries	---	1.2E-01	---	1.8E-01	---	1.8E-01	---	5.8E-02
Pancreas	7.9E-02	7.9E-02	2.1E-01	2.0E-01	2.1E-01	2.0E-01	3.3E-02	3.3E-02
Prostate	1.4E-01	---	2.4E-01	---	2.4E-01	---	6.1E-02	---
Red (active) bone marrow	6.8E-02	6.8E-02	1.7E-01	1.7E-01	1.7E-01	1.7E-01	2.9E-02	2.9E-02
Salivary glands	5.2E-02	5.2E-02	1.5E-01	1.5E-01	1.5E-01	1.5E-01	2.7E-02	2.7E-02
Skin	4.2E-02	4.2E-02	1.1E-01	1.1E-01	1.1E-01	1.1E-01	1.6E-02	1.6E-02
Small intestine wall	6.3E-02	6.3E-02	1.4E-01	1.4E-01	1.4E-01	1.4E-01	2.3E-02	2.4E-02
Spleen	7.0E-02	7.0E-02	1.7E-01	1.7E-01	1.7E-01	1.7E-01	2.8E-02	2.8E-02
Stomach wall	5.8E-02	5.8E-02	1.3E-01	1.3E-01	1.3E-01	1.3E-01	2.2E-02	2.2E-02
Testes	5.5E-02	---	1.3E-01	---	1.3E-01	---	2.9E-02	---
Thymus	6.9E-02	7.0E-02	1.7E-01	1.7E-01	1.7E-01	1.7E-01	2.6E-02	2.6E-02
Thyroid	5.3E-02	5.3E-02	1.4E-01	1.4E-01	1.4E-01	1.4E-01	2.3E-02	2.3E-02
Urinary bladder wall	2.7E-01	2.7E-01	3.9E-01	3.9E-01	3.9E-01	3.9E-01	1.6E-01	1.6E-01
Uterus/cervix	---	3.3E-01	---	3.8E-01	---	3.8E-01	---	1.1E-01
Effective dose (publ. 60) [mSv/MBq]	7.9E-02	9.2E-02	1.7E-01	1.8E-01	1.7E-01	1.8E-01	4.6E-03	4.6E-03
Effective dose (publ. 103) [mSv/MBq]	7.5E-02		1.6E-01		1.6E-01		3.4E-02	
Detriment adjusted radiation risk* (publ. 103) [mSv/MBq]	7.2E-02	7.9E-02	1.6E-01	1.6E-01	1.6E-01	1.6E-01	3.2E-02	3.5E-02

*Detriment adjusted radiation risk is non-sex averaging effective dose, e.g. sex specific effective dose

Data in Table 1b are given in mGy/MBq unless otherwise noted. Effective dose was calculated using the two sets of tissue weighting factors given in ICRP Publications 60 and 103

The absorbed dose and effective dose for oral administration of ^99m^Tc-pertechnetate are presented in Table 2. For oral administration of ^99m^Tc-pertechnetate, the highest absorbed dose was received by the thyroid for all 14 reference individuals and phantoms. The effective dose per unit administered activity for adult, 15- 10-, 5-, 1-year, 100 days (infant) and 0 day (newborn) for ^99m^Tc-pertechnetate, using the tissue weighting factors from ICRP Publication 103, were calculated to 0.013 mSv/MBq, 0.017 mSv/MBq, 0.024 mSv/MBq, 0.040 mSv/MBq, 0.082 mSv/MBq 0.16 mSv/MBq and 0.16 mSv/MBq, respectively. The interpolation for male phantoms could be performed up to 73 kg, for ^131^I-iodide, were an “interpolated” individual of total male weight of a 68 kg calculated and the effective dose per unit administered activity to 0.041 mSv/MBq.

**Table 2a:** Absorbed dose per unit of orally administered ^99m^Tc-pertechnetate activity calculated with IDAC-Dose2.2

Organ	^99m^Tc-pertechnetate							
	Adult		15 years old		10 years old		5 years old	
	Male	Female	Male	Female	Male	Female	Male	Female
Adrenals	7.0E-03	7.5E-03	5.6E-03	5.9E-03	8.5E-03	8.5E-03	1.3E-02	1.3E-02
Brain	2.2E-03	2.8E-03	4.3E-03	4.4E-03	6.4E-03	6.5E-03	9.1E-03	9.2E-03
Breast	2.3E-03	2.4E-03	2.4E-03	2.7E-03	3.7E-03	3.3E-03	6.0E-03	5.8E-03
Colon wall	3.6E-02	4.2E-02	4.5E-02	4.8E-02	6.6E-02	6.5E-02	1.0E-01	1.0E-01
Endosteum (bone surface)	7.5E-03	9.9E-03	1.1E-02	1.1E-02	1.3E-02	1.3E-02	2.5E-02	2.5E-02
ET region	2.2E-03	3.2E-03	6.0E-03	5.8E-03	7.6E-03	7.5E-03	9.5E-03	9.5E-03
Eye lenses	1.4E-03	1.8E-03	3.6E-03	3.8E-03	5.5E-03	5.5E-03	6.8E-03	6.7E-03
Gallbladder wall	1.0E-02	6.1E-03	6.4E-03	7.4E-03	1.0E-02	1.0E-02	1.7E-02	1.7E-02
Heart wall	6.0E-03	6.2E-03	3.5E-03	4.1E-03	6.0E-03	6.0E-03	9.5E-03	9.5E-03
Kidneys	1.1E-02	1.2E-02	9.3E-03	9.8E-03	1.4E-02	1.4E-02	2.2E-02	2.2E-02
Liver	7.2E-03	6.4E-03	5.9E-03	7.0E-03	9.7E-03	9.7E-03	1.5E-02	1.5E-02
Lungs	4.5E-03	5.2E-03	4.6E-03	5.2E-03	7.1E-03	7.1E-03	1.1E-02	1.1E-02
Lymphatic nodes	6.5E-03	7.6E-03	6.3E-03	6.8E-03	9.0E-03	9.0E-03	1.5E-02	1.5E-02
Muscle	3.2E-03	4.2E-03	3.6E-03	3.5E-03	5.3E-03	5.3E-03	8.1E-03	8.0E-03
Oesophagus	5.8E-03	6.3E-03	5.8E-03	6.2E-03	9.1E-03	9.1E-03	1.4E-02	1.4E-02
Oral mucosa	2.6E-03	3.4E-03	6.2E-03	6.5E-03	7.9E-03	7.9E-03	9.0E-03	9.0E-03
Ovaries	---	1.2E-02	---	1.9E-02	---	2.4E-02	---	3.6E-02
Pancreas	1.1E-02	9.9E-03	7.8E-03	7.9E-03	1.1E-02	1.1E-02	1.6E-02	1.6E-02
Prostate	1.2E-02	---	1.3E-02	---	2.2E-02	---	3.1E-02	---
Red (active) bone marrow	6.8E-03	9.4E-03	8.5E-03	9.3E-03	1.2E-02	1.1E-02	1.8E-02	1.7E-02
Salivary glands	9.9E-03	1.3E-02	1.6E-02	1.6E-02	2.2E-02	2.2E-02	2.7E-02	2.7E-02
Skin	2.1E-03	2.6E-03	2.6E-03	2.8E-03	4.3E-03	4.3E-03	6.8E-03	6.8E-03
Small intestine wall	1.0E-02	1.3E-02	1.1E-02	1.1E-02	1.5E-02	1.6E-02	2.6E-02	2.7E-02
Spleen	5.8E-03	6.8E-03	5.0E-03	6.0E-03	8.2E-03	8.2E-03	1.4E-02	1.4E-02
Stomach wall	1.4E-02	1.5E-02	1.5E-02	1.6E-02	2.3E-02	2.3E-02	3.5E-02	3.5E-02
Testes	2.9E-03	---	8.1E-03	---	9.3E-03	---	1.5E-02	---
Thymus	3.5E-03	3.9E-03	4.4E-03	5.1E-03	6.3E-03	6.3E-03	9.9E-03	9.9E-03
Thyroid	5.7E-02	6.9E-02	9.2E-02	9.6E-02	1.4E-01	1.4E-01	3.1E-01	3.1E-01
Urinary bladder wall	1.0E-02	1.3E-02	1.3E-02	1.3E-02	1.9E-02	1.9E-02	2.5E-02	2.5E-02
Uterus/cervix	---	1.5E-02	---	5.0E-02	---	6.8E-02	---	4.1E-02
Effective dose (publ. 60) [mSv/MBq]	1.2E-02	1.6E-02	1.7E-02	2.0E-02	2.4E-02	2.7E-02	4.2E-02	4.7E-02
Effective dose (publ. 103) [mSv/MBq]	1.3E-02		1.7E-02		2.4E-02		4.0E-02	
Detriment adjusted radiation dose* (publ. 103) [mSv/MBq]	1.2E-02	1.5E-02	1.5E-02	1.8E-02	2.3E-02	2.4E-02	3.9E-02	4.1E-02

*Detriment adjusted radiation risk is non-sex averaging effective dose, e.g. sex specific effective dose

Data in Table 2a are given in mGy/MBq unless otherwise noted. Effective dose was calculated using the two sets of tissue weighting factors given in ICRP Publications 60 and 103

**Table 2b:** Absorbed dose per unit of orally administered ^99m^Tc-pertechnetate activity calculated with IDAC-Dose2.2

Organ	^99m^Tc-pertechnetate							
	1 year old		Infant (100 days)		Newborn (0 day)		Interpol. 68 kg male	
	Male	Female	Male	Female	Male	Female	Male	Female
Adrenals	2.5E-02	2.5E-02	4.9E-02	4.9E-02	4.9E-02	4.9E-02	1.3E-02	1.3E-02
Brain	1.4E-02	1.4E-02	3.3E-02	3.3E-02	3.3E-02	3.3E-02	9.3E-03	9.4E-03
Breast	1.0E-02	1.0E-02	2.6E-02	2.5E-02	2.6E-02	2.5E-02	6.1E-03	6.0E-03
Colon wall	2.5E-01	2.4E-01	5.8E-01	5.8E-01	5.8E-01	5.8E-01	1.1E-01	1.1E-01
Endosteum (bone surface)	5.2E-02	5.2E-02	8.5E-02	8.4E-02	8.5E-02	8.4E-02	2.6E-02	2.6E-02
ET region	1.4E-02	1.4E-02	3.2E-02	3.2E-02	3.2E-02	3.2E-02	9.7E-03	9.6E-03
Eye lenses	9.6E-03	9.6E-03	2.7E-02	2.7E-02	2.7E-02	2.7E-02	6.8E-03	6.8E-03
Gallbladder wall	3.0E-02	3.0E-02	7.2E-02	7.2E-02	7.2E-02	7.2E-02	1.7E-02	1.7E-02
Heart wall	1.7E-02	1.7E-02	3.8E-02	3.8E-02	3.8E-02	3.8E-02	9.7E-03	9.7E-03
Kidneys	3.6E-02	3.6E-02	7.9E-02	7.8E-02	7.9E-02	7.8E-02	2.3E-02	2.3E-02
Liver	2.7E-02	2.7E-02	5.8E-02	5.8E-02	5.8E-02	5.8E-02	1.5E-02	1.5E-02
Lungs	2.2E-02	2.2E-02	4.8E-02	4.8E-02	4.8E-02	4.8E-02	1.1E-02	1.1E-02
Lymphatic nodes	2.6E-02	2.6E-02	4.4E-02	4.4E-02	4.4E-02	4.4E-02	1.5E-02	1.5E-02
Muscle	1.5E-02	1.4E-02	3.6E-02	3.6E-02	3.6E-02	3.6E-02	8.3E-03	8.2E-03
Oesophagus	2.4E-02	2.4E-02	5.1E-02	5.1E-02	5.1E-02	5.1E-02	1.5E-02	1.5E-02
Oral mucosa	1.4E-02	1.4E-02	4.4E-02	4.4E-02	4.4E-02	4.4E-02	9.1E-03	9.1E-03
Ovaries	---	6.3E-02	---	8.2E-02	---	8.2E-02	---	3.7E-02
Pancreas	2.9E-02	2.9E-02	5.5E-02	5.5E-02	5.5E-02	5.5E-02	1.6E-02	1.6E-02
Prostate	6.0E-02	---	9.9E-02	---	9.9E-02	---	3.2E-02	---
Red (active) bone marrow	3.3E-02	3.3E-02	8.3E-02	8.3E-02	8.3E-02	8.3E-02	1.8E-02	1.8E-02
Salivary glands	3.9E-02	3.9E-02	1.4E-01	1.4E-01	1.4E-01	1.4E-01	2.8E-02	2.8E-02
Skin	1.2E-02	1.2E-02	3.0E-02	3.0E-02	3.0E-02	3.0E-02	7.0E-03	7.0E-03
Small intestine wall	4.7E-02	4.7E-02	8.3E-02	8.1E-02	8.3E-02	8.1E-02	2.7E-02	2.8E-02
Spleen	2.5E-02	2.5E-02	5.2E-02	5.2E-02	5.2E-02	5.2E-02	1.4E-02	1.4E-02
Stomach wall	7.5E-02	7.5E-02	1.7E-01	1.7E-01	1.7E-01	1.7E-01	3.6E-02	3.6E-02
Testes	2.0E-02	---	3.8E-02	---	3.8E-02	---	1.5E-02	---
Thymus	2.0E-02	2.0E-02	4.3E-02	4.3E-02	4.3E-02	4.3E-02	1.0E-02	1.0E-02
Thyroid	5.9E-01	5.9E-01	7.9E-01	7.9E-01	7.9E-01	7.9E-01	3.2E-01	3.2E-01
Urinary bladder wall	3.9E-02	3.9E-02	5.9E-02	5.6E-02	5.9E-02	5.6E-02	2.6E-02	2.6E-02
Uterus/cervix	---	1.7E-01	---	1.9E-01	---	1.9E-01	---	4.2E-02
Effective dose (publ. 60) [mSv/MBq]	8.5E-02	9.3E-02	1.7E-01	1.7E-01	1.7E-01	1.7E-01	4.5E-04	4.5E-04
Effective dose (publ. 103) [mSv/MBq]	8.2E-02		1.6E-01		1.6E-01		4.1E-02	
Detriment adjusted radiation dose* (publ. 103) [mSv/MBq]	8.0E-02	8.4E-02	1.6E-01	1.6E-01	1.6E-01	1.6E-01	4.0E-02	4.2E-02

*Detriment adjusted radiation risk is non-sex averaging effective dose, e.g. sex specific effective dose

Data in Table 2b are given in mGy/MBq unless otherwise noted. Effective dose was calculated using the two sets of tissue weighting factors given in ICRP Publications 60 and 103

The absorbed dose and effective dose for oral administration of ^131^I-iodide are presented in Table 3. The presented absorbed doses in Tables 1, 2 and 3 for 2 -[^18^F]FDG, ^99m^Tc-pertechnetate and ^131^I-iodide are given for the target organs given a tissue weighting factor in ICRP Publication 103 together with absorbed dose data for the lens of the eye. For oral administration with medium thyroid uptake of ^131^I-iodide, the highest absorbed dose for all 12 phantoms was received by the thyroid. The effective dose per unit administered activity for adult, 15- 10-, 5-, 1-year, 100 days (infant) and 0 day (newborn) for ^131^I-iodide, using the tissue weighting factors from ICRP Publication 103, was calculated to 16 mSv/MBq, 24 mSv/MBq, 35 mSv/MBq, 74 mSv/MBq, 120 mSv/MBq 140 mSv/MBq and 140 mSv/MBq respectively. The effective dose per unit administered activity was also calculated to 100 mSv/MBq for a “interpolated” individual of total female weight of 45 kg representing an 8-years-old reference individual. Instead of assuming a 3.5 h voiding interval with a static urinary bladder, the sub-module for a dynamic urinary bladder was applied to the ^131^I-iodide model. An additional calculation was performed with a dynamic urinary bladder model applied to the adult male biokinetic model of medium thyroid uptake of orally administered ^131^I-iodide. The dynamic parameters are shown in Fig. 2 and the data shows that for this case the cumulated activities for the urinary bladder content will be 14% (1.14 MBq-h/MBq /1.31 MBq-h/MBq) lower compared to the 3.5 h voiding interval assumption.

## Discussion

For the three radiopharmaceuticals 2-[^18^F]FDG, ^99m^Tc-pertechnetate and ^131^Iodide absorbed doses have been calculated using IDAC-Dose2.2 to all 12 ICRP reference individuals and the 0-day computational phantom. The absorbed doses are presented for organs and tissues as well as effective doses using weighting factors from ICRP Publication 103 [12]. The absorbed dose to the lens of the eye is also given.

For oral administration with medium thyroid uptake of ^131^I-iodide, the effective dose in ICRP Publication 128 [26] was estimated to be 53 mSv/MBq for a 10-year-old and, in this paper, using new tissue weighting factors, new decay scheme and SAF values, the coefficient was estimated to be 35 mSv/MBq, which corresponds to a 39% reduction compared to ICRP Publication 128. In general, is it not possible to determine explicitly which factor contributes most to the change in the coefficient. However, in the case for ^131^I-iodide where 98.9% of effective dose value arises from irradiation of the thyroid the dominating factor for the effective dose reduction is the change of the tissue weighting factor for the thyroid from 0.05 in ICRP Publication 60 to 0.04 in ICRP Publication 103.

For the orally administered ^131^I-iodid, biokinetic model with medium thyroid uptake the urinary bladder content will in most cases be estimated to be larger than using the static volume, which means that for homogeneously distributed activity there will be a lower energy deposition in surrounding organs and tissues. Both these factors will decrease the absorbed dose to the urinary bladder wall.

The updated ICRP framework for internal dose assessment for reference phantoms deviates from the RADAR (Radiation Dose Assessment Resource) method and the MIRD (Medical Internal Radiation Dose) method for internal dose estimation, the two later methods of internal dose calculations are conceptually the same [36]. One of the main differences in principle is how the circulating blood is treated. The RADAR and MIRD is based on descriptive biokinetic modelling, where the activity in each organ is assumed as a separate entity, with an instant organ uptake and retention without any transfer of decays in the circulating blood. The background activity in the source region “Other” (called “Remainder” in MIRD and RADAR) is distributed through the whole body. The ICRP framework for internal dose assessment for reference phantoms is defined for systemic compartment modelling where the circulating blood is the central compartment for transfer of radionuclides between different organs and tissues, and therefore is there a specific source region for the circulating blood, which is distributed thought the entire phantom. The background activity in source region “Other” is in the ICRP model only distributed to organs and tissue with connection and transfer with the circulating blood e.g. the contents in organs or the air in the body are not in this transfer nor in the source region Other. For dosimetric calculation the ICRP crates a $\:S$-values based on the whole beta spectrum while the other methods use the mean beta energy. Due to differences in internal dose methods the absorbed dose and effective dose will deviate depending on which framework/method is used.

The absorbed dose and effective dose for the current 2-[^18^F]FDG model presented in ICRP Publ. 128 [27] were compared with the RADAR software OLINDA-EXE version 2.0 [36], which is based on another set of SAF-values but also based on the anatomical and physiological data for use in Radiological Protection [10,36]. For the assessment of effective dose for 2-[^18^F]FDG were the quotient (IDAC-Dose2.2/OLINDA-EXE2.0) for adults, 15, 10, 5 and 1-year old are 0.84 (1.61E-02/1.92E-02), 0.88 (1.94E-02/2.20E-02), 0.88 (2.84E-02/3.23E-02), 0.90 (4.34E-02/4.82E-02) and 0.97 (7.80E-02/8.01E-02), respectively. OLINDA-EXE2.0 do not strictly follow the ICRP scheme to calculate effective dose [12], as it excludes the blood of the target regions [22]. This modification will be the most contributing factor when investigating the differences in absorbed doses between the two different software codes. E.g. the ICRP reference target mass of the liver is 2.36 kg [22] and by removing the blood from this reference organ, the liver mass decreases to 1.8 kg. As absorbed dose is energy absorbed divided the mass of the organ the liver absorbed dose will always be a factor of 1.3 (2.36 kg/1.8 kg) higher in OLINDA-EXE2.0 regardless of previous mentioned differences between the ICRP computational framework and RADAR/MIRD methods. No absorbed dose comparisons between the IDAC-Dose2.2 and OLINDA/EXE2.0 were performed on the technetium, the revised FGD or the iodide models given in Tables 1, 2 and 3. The technetium, the revised FDG and the iodide models are systemic compartment models assuming transfer between organs and tissues of radionuclides of transfer into the circulating blood, and as the MIRD method do not treat the circulating blood as a separate source region therefore this comparison was not possible. A comprehensive comparison between OLINDA/EXE2.0 and the adult phantoms of IDAC-Dose has been made by MIRD [37]. The comparison shows the difference between OLINDA and IDAC-Dose and that the adult phantoms of IDAC-Dose and MIRD software MIRDcalc have a good agreement. IDAC-Dose2.2 retains the computational methodology of its predecessor, IDAC-Dose2.1; however, it constitutes a significant advancement in internal dosimetry by enabling dose calculations across 12 standardized computational phantoms, as opposed to only 2 in the earlier version. This expansion enhances the anatomical diversity represented in dose assessments, thereby increasing the robustness and applicability of the software for radiological protection and individualized dosimetric evaluations. The comparison and conclusion made by MIRD [37] is also valid for IDAC-Dose2.2.

However, the main uncertainty lies in the biokinetic models used. All biokinetic models are a rough mathematical description of complex biological and physiological processes for which there is limited knowledge. The ICRP models are extracted from an often-limited series of human and sometimes animal data through judgements [27,38].

The software offers the option of calculating the absorbed dose to 48 different organs and tissues. The tissue weighting factor is defined to reflect the relative contribution of the organ and tissue to the detriment for stochastic effects.

**Table 3a:** Absorbed dose per unit of orally administered ^131^I-iodide uptake activity calculated with IDAC-Dose2.2

Organ	^131^I-iodide (with medium thyroid uptake)							
	Adult		15 years old		10 years old		5 years old	
	Male	Female	Male	Female	Male	Female	Male	Female
Adrenals	8.3E-02	9.4E-02	7.8E-02	8.5E-02	1.3E-01	1.3E-01	2.4E-01	2.4E-01
Brain	5.9E-02	8.9E-02	9.1E-02	1.0E-01	1.7E-01	1.7E-01	2.2E-01	2.2E-01
Breast	6.1E-02	1.4E-01	5.8E-02	7.2E-02	1.1E-01	1.1E-01	1.9E-01	1.9E-01
Colon wall	6.3E-02	7.1E-02	7.7E-02	7.4E-02	1.2E-01	1.2E-01	2.1E-01	2.1E-01
Endosteum (bone surface)	9.9E-02	1.3E-01	1.2E-01	1.3E-01	1.9E-01	1.9E-01	3.7E-01	3.7E-01
ET region	5.5E-01	8.2E-01	2.9E-01	3.6E-01	4.9E-01	4.9E-01	5.8E-01	5.8E-01
Eye lenses	6.0E-02	9.7E-02	8.9E-02	1.1E-01	1.8E-01	1.8E-01	2.2E-01	2.2E-01
Gallbladder wall	7.2E-02	8.2E-02	7.9E-02	9.2E-02	1.1E-01	1.1E-01	2.0E-01	2.0E-01
Heart wall	2.3E-01	2.4E-01	2.1E-01	2.2E-01	2.6E-01	2.6E-01	4.2E-01	4.2E-01
Kidneys	1.9E-01	2.2E-01	2.3E-01	2.6E-01	3.7E-01	3.7E-01	7.0E-01	7.0E-01
Liver	1.3E-01	1.6E-01	1.6E-01	1.8E-01	2.9E-01	2.9E-01	5.4E-01	5.4E-01
Lungs	2.9E-01	3.3E-01	9.9E-01	8.4E-01	9.9E-01	9.9E-01	1.7E + 00	1.7E + 00
Lymphatic nodes	9.1E-01	9.8E-01	3.0E-01	3.2E-01	3.3E-01	3.3E-01	6.1E-01	6.1E-01
Muscle	8.9E-02	1.3E-01	8.1E-02	7.9E-02	1.3E-01	1.3E-01	2.5E-01	2.6E-01
Oesophagus	2.2E + 00	2.5E + 00	1.0E + 00	1.1E + 00	1.2E + 00	1.2E + 00	2.3E + 00	2.4E + 00
Oral mucosa	2.2E-01	5.0E-01	5.7E-01	6.8E-01	5.1E-01	5.1E-01	6.2E-01	6.2E-01
Ovaries	---	6.8E-02	---	1.0E-01	---	1.5E-01	---	2.3E-01
Pancreas	7.6E-02	8.3E-02	7.9E-02	8.5E-02	1.3E-01	1.3E-01	2.2E-01	2.2E-01
Prostate	6.3E-02	---	7.7E-02	---	1.4E-01	---	2.2E-01	---
Red (active) bone marrow	2.0E-01	2.4E-01	1.8E-01	1.9E-01	2.4E-01	2.4E-01	3.8E-01	3.8E-01
Salivary glands	4.4E-01	6.9E-01	6.2E-01	7.0E-01	1.3E + 00	1.3E + 00	1.5E + 00	1.5E + 00
Skin	5.7E-02	7.2E-02	6.7E-02	7.8E-02	1.1E-01	1.1E-01	1.8E-01	1.8E-01
Small intestine wall	5.4E-02	6.9E-02	5.9E-02	6.3E-02	9.4E-02	9.6E-02	1.8E-01	1.8E-01
Spleen	9.0E-02	9.9E-02	7.2E-02	8.8E-02	1.4E-01	1.4E-01	2.5E-01	2.5E-01
Stomach wall	3.5E-01	3.6E-01	4.4E-01	4.7E-01	6.9E-01	6.9E-01	1.0E + 00	1.0E + 00
Testes	2.1E-02	---	5.0E-02	---	7.3E-02	---	1.3E-01	---
Thymus	2.4E + 00	2.1E + 00	2.0E + 00	3.1E + 00	1.5E + 00	1.5E + 00	2.1E + 00	2.1E + 00
Thyroid	3.6E + 02	4.4E + 02	5.9E + 02	6.1E + 02	8.7E + 02	8.7E + 02	1.8E + 03	1.8E + 03
Urinary bladder wall	1.7E-01	2.2E-01	2.2E-01	2.3E-01	3.8E-01	3.8E-01	5.2E-01	5.2E-01
Uterus/cervix	---	8.5E-02	---	2.7E-01	---	4.0E-01	---	2.7E-01
Effective dose (publ. 60) [mSv/MBq]	1.8E + 01	2.2E + 01	3.0E + 01	3.1E + 01	4.4E + 01	4.4E + 01	9.3E + 01	9.3E + 01
Effective dose (publ. 103) [mSv/MBq]	1.6E + 01		2.4E + 01		3.5E + 01		7.4E + 01	
Detriment adjusted radiation dose* (publ. 103) [mSv/MBq]	1.5E + 01	1.8E + 01	2.4E + 01	2.5E + 01	3.5E + 01	3.5E + 01	7.4E + 01	7.4E + 01

*Detriment adjusted radiation risk is non-sex averaging effective dose, e.g. sex specific effective dose

Data in Table 3a are given in mGy/MBq unless otherwise noted. Effective dose was calculated using the two sets of tissue weighting factors given in ICRP Publications 60 and 103

**Table 3b:** Absorbed dose per unit of orally administered ^131^I-iodide uptake activity calculated with IDAC-Dose2.2

Organ	^131^I-iodide (with medium thyroid uptake)							
	1 year old		Infant (100 days)		Newborn (0 day)		Interpol.45 kg female	
	Male	Female	Male	Female	Male	Female	Male	Female
Adrenals	6.0E-01	6.0E-01	1.2E + 00	1.2E + 00	1.2E + 00	1.2E + 00	4.4E-01	4.4E-01
Brain	3.7E-01	3.7E-01	1.1E + 00	1.1E + 00	1.1E + 00	1.1E + 00	3.0E-01	3.0E-01
Breast	3.7E-01	3.7E-01	9.1E-01	9.1E-01	9.1E-01	9.1E-01	2.9E-01	2.9E-01
Colon wall	5.0E-01	4.9E-01	1.2E + 00	1.2E + 00	1.2E + 00	1.2E + 00	3.7E-01	3.7E-01
Endosteum (bone surface)	7.7E-01	7.7E-01	1.9E + 00	1.9E + 00	1.9E + 00	1.9E + 00	6.0E-01	6.0E-01
ET region	6.3E-01	6.3E-01	2.8E + 00	2.8E + 00	2.8E + 00	2.8E + 00	6.1E-01	6.1E-01
Eye lenses	2.9E-01	2.9E-01	1.0E + 00	1.0E + 00	1.0E + 00	1.0E + 00	2.6E-01	2.6E-01
Gallbladder wall	4.4E-01	4.4E-01	1.0E + 00	1.0E + 00	1.0E + 00	1.0E + 00	3.3E-01	3.4E-01
Heart wall	8.8E-01	8.8E-01	1.7E + 00	1.7E + 00	1.7E + 00	1.7E + 00	6.8E-01	6.8E-01
Kidneys	1.6E + 00	1.6E + 00	4.4E + 00	4.4E + 00	4.4E + 00	4.4E + 00	1.2E + 00	1.2E + 00
Liver	1.4E + 00	1.4E + 00	3.5E + 00	3.5E + 00	3.5E + 00	3.5E + 00	1.0E + 00	1.0E + 00
Lungs	4.4E + 00	4.4E + 00	3.4E + 00	3.4E + 00	3.4E + 00	3.4E + 00	3.2E + 00	3.2E + 00
Lymphatic nodes	9.3E-01	9.3E-01	1.7E + 00	1.7E + 00	1.7E + 00	1.7E + 00	8.1E-01	8.1E-01
Muscle	5.1E-01	5.1E-01	1.5E + 00	1.5E + 00	1.5E + 00	1.5E + 00	4.0E-01	4.0E-01
Oesophagus	4.6E + 00	4.6E + 00	4.7E + 00	4.7E + 00	4.7E + 00	4.7E + 00	3.7E + 00	3.7E + 00
Oral mucosa	6.7E-01	6.7E-01	4.4E + 00	4.4E + 00	4.4E + 00	4.4E + 00	6.5E-01	6.6E-01
Ovaries	---	4.4E-01	---	8.9E-01	---	8.9E-01	---	3.5E-01
Pancreas	4.6E-01	4.6E-01	1.1E + 00	1.1E + 00	1.1E + 00	1.1E + 00	3.6E-01	3.6E-01
Prostate	4.3E-01	---	8.3E-01	---	8.3E-01	---	3.4E-01	---
Red (active) bone marrow	7.4E-01	7.4E-01	1.9E + 00	1.9E + 00	1.9E + 00	1.9E + 00	5.8E-01	5.8E-01
Salivary glands	2.0E + 00	2.0E + 00	8.3E + 00	8.3E + 00	8.3E + 00	8.3E + 00	1.8E + 00	1.8E + 00
Skin	3.1E-01	3.1E-01	8.4E-01	8.4E-01	8.4E-01	8.4E-01	2.6E-01	2.6E-01
Small intestine wall	4.1E-01	4.1E-01	1.0E + 00	1.0E + 00	1.0E + 00	1.0E + 00	3.1E-01	3.1E-01
Spleen	5.9E-01	5.9E-01	1.3E + 00	1.3E + 00	1.3E + 00	1.3E + 00	4.4E-01	4.4E-01
Stomach wall	2.1E + 00	2.1E + 00	3.5E + 00	3.5E + 00	3.5E + 00	3.5E + 00	1.6E + 00	1.6E + 00
Testes	2.2E-01	---	5.7E-01	---	5.7E-01	---	1.8E-01	---
Thymus	5.3E + 00	5.3E + 00	4.6E + 00	4.6E + 00	4.6E + 00	4.6E + 00	3.9E + 00	3.9E + 00
Thyroid	3.0E + 03	3.0E + 03	3.4E + 03	3.4E + 03	3.4E + 03	3.4E + 03	2.6E + 03	2.6E + 03
Urinary bladder wall	8.0E-01	7.9E-01	1.0E + 00	9.9E-01	1.0E + 00	9.9E-01	6.8E-01	6.8E-01
Uterus/cervix	---	1.2E + 00	---	1.9E + 00	---	1.9E + 00	---	7.6E-01
Effective dose (publ. 60) [mSv/MBq]	1.5E + 02	1.5E + 02	1.7E + 02	1.7E + 02	1.7E + 02	1.7E + 02	2.1E-02	2.1E-02
Effective dose (publ. 103) [mSv/MBq]	1.2E + 02		1.4E + 02		1.4E + 02		1.0E + 02	
Detriment adjusted radiation dose* (publ. 103) [mSv/MBq]	1.2E + 02	1.2E + 02	1.4E + 02	1.4E + 02	1.4E + 02	1.4E + 02	1.0E + 02	1.0E + 02

*Detriment adjusted radiation risk is non-sex averaging effective dose, e.g. sex specific effective dose

Data in Table 3b are given in mGy/MBq unless otherwise noted. Effective dose was calculated using the two sets of tissue weighting factors given in ICRP Publications 60 and 103

## Conclusions

IDAC-dose2.2 uses the latest ICRP computational framework for internal dose assessment for reference phantoms. This framework emphasizes that representation of the circulating blood and the ICRP has thus changed from descriptive biokinetic models to systemic biokinetic compartment models, making them more physiologically realistic representations of uptake and retention in organs and tissues, and excretion [1]. The IDAC-Dose2.2 was developed for the ICRP Task Group Radiation Dose to Patients in Diagnostic Nuclear Medicine and the software will be used for the dose calculations in the upcoming revised version of ICRP Publication 128.

IDAC-Dose2.2 is an updated version of IDAC-Dose2.1, the new version includes absorbed does calculations to newborn, infants, 1 year old, 5 years old, 10 years old and 15 years old. The is also a possibility to interpolate the absorbed doses between phantoms either by weight or age.

Absorbed dose and effective dose calculations were performed for all twelve ICRP computational reference phantoms, using the latest biokinetic models for 2-[^18^F]FDG, ^99m^Tc-pertechnetate, and ^131^I-iodide. The S-values of IDAC-Dose2.2 was validated against DCAL ver. 2022 with identical results. The two ICRP programs have been harmonised and based their calculations on the same computational framework, so that identical radiation exposures give the same absorbed dose independent of the situation for which they are estimated. IDAC-Dose2.2 is used by the ICRP for absorbed and effective dose calculations in diagnostic nuclear medicine.

The IDAC-Dose2.2 program is free software for research and available at www.idac-dose.org. The online version can be operated directly through a web browser and the standalone version is an executable file, which is downloaded and installed directly on the local computer.

## Data Availability

The datasets used and/or analysed during the current study are available from the corresponding author on reasonable request.

## Abbreviations

IDAC: Internal Dose Assessed by Computer
DCAL: Dose and risk calculation
ICRP: International Commission on Radiological Protection
SAF: Specific absorbed fraction
FDG: Fluorodeoxyglucose, 2-deoxy-2-fluoro-D-glucose
